# Supplementary material for: Improvements in Maturity and Stability of 3D iPSC-Derived Hepatocyte-like Cell Cultures
Source: Cells. 2023 Sep 27;12(19):2368. doi: 10.3390/cells12192368 (PMC10571736; doi:10.3390/cells12192368)
Supplement: Supplementary file 1 [file cells-12-02368-s001.zip › Supplementary methods, tables and figures.pdf]

Improvements in maturity and stability of 3D iPSC-derived hepatocyte-like cell cultures

Siiri Suominen[1,*,] Tinja Hyypijev[1,†], Mari Venäläinen[1,†], Alma Yrjänäinen[2,3], Hanna Vuorenpää[2,3], Mari Lehti-Polojärvi[4], Mikko Räsänen[5], Aku Seppänen[5], Jari Hyttinen[4], Susanna Miettinen[2,3] and Katriina Aalto-Setälä[1,6] & Leena E. Viiri[1]

[1] Heart Group, Finnish Cardiovascular Research Center and Science mimicking life Research Center, Faculty of Medicine and Health Technology, Tampere University, 33520 Tampere, Finland.

[2] Adult Stem Cell Group, Faculty of Medicine and Health Technology, 33520 Tampere University.

[3]Research, Development and Innovation Centre, Tampere University Hospital, 33520 Tampere, Finland.

[4]Computational Biophysics and Imaging Group, Faculty of Medicine and Health Technology, Tampere University, 33520 Tampere, Finland

[5]Department of Technical Physics, University of Eastern Finland, 70210 Kuopio, Finland.

[6]Heart Hospital, Tampere University Hospital, 33520 Tampere, Finland.

[†] These authors contributed equally.

* Correspondence: siiri.suominen@tuni.fi

Supplementary methods

Immunocytochemical staining of 2D iPSC-HLC cultures

Protein expression of liver-specific markers in 2D control cultures was determined with immunocytochemical staining, according to the protocol presented on Table S3. The antibodies and their dilutions are presented in Table S4. Samples were fixed with 4% paraformaldehyde (PFA) prior to staining. All longer incubations were done on a plate shaker. Stained samples were dried and mounted with Vectashield Antifade Mounting Medium with DAPI (Vector Laboratories, Newark, CA, USA) and covered with coverslips. Stained samples were imaged with Olympus IX51 Inverted Phase Contrast Fluorescence Microscope (Olympus Corporation, Hamburg, Germany).

Immunohistochemical analysis of spheroid sections

Protein expression of liver-specific markers in the spheroids was determined by immunohistochemically staining sections of the spheroids. Spheroid droplets were prepared by washing with 2x PBS, incubating 5 minutes per wash, and then fixing with 4% PFA for 25–30 minutes. After fixing, samples were washed 3x PBS with 10-minute incubation for each wash. Fixed samples were stored at +4°C. Two spheroids per time point were chosen for further processing. These fixed samples were dehydrated with KEDEE Tissue Processor and then embedded in paraffin. Paraffin

blocks were cut to 4 μm sections with Leica SM2010 R microtome and collected to charged slides. Around 20–30 sections from the middle part of the spheroids were cut and four successful sections per time point were chosen for staining. Spheroid sections were dried upward at room temperature overnight. Antigen retrieval was done by first incubating the sections at +60°C for an hour. Incubated sections were dewaxed using Stainmate (Thermo Scientific) device. Dewaxed sections were moved to pH 9 Tris-EDTA buffer (Tris: 0.05 M, EDTA 0.001 M; diluted in 0.05% Tween 20) and heated at +121°C for 2 minutes. Samples were then moved to a staining rack in TBS+0.05% Tween 20 buffer. Tween 20 was used instead of PBS to promote more effective washing and prevent non-specific staining.

Biomaterial preparation for chip experiments

The fibrinogen solution was prepared a day before use by dissolving fibrinogen from human plasma (Sigma-Aldrich) in warm phosphate buffered saline (PBS, Lonza). A 100 U/ml thrombin stock solution was prepared by dissolving thrombin from human plasma (Merck Millipore) in 0.1% bovine serum albumin (BSA, Sigma-Aldrich) in sterile water. The stock solution was further diluted to a working solution of 2 IU/ml before use. Fibrinogen and thrombin were mixed in 1:1 ratio, making the final thrombin concentration in the gel 1 IU/ml and the fibrinogen concentrations 10, 5.0 and 2.5 mg/ml.

To find an optimal collagen gel composition for the chip experiments with hepatocytes, three different collagen I concentrations were tested: 2.0, 1.0 and 0.5 mg/ml. The collagen mixtures were prepared on ice by mixing 3 mg/ml collagen I (Gibco) with 10XPBS (Gibco), sodium hydroxide (NaOH) and sterile water to reach the desired concentration and volume. After mixing the components, the pH of the mixture was checked with a pH indicator paper to make sure it was within the appropriate range of 6.5–7.5. The mixtures were kept on ice to avoid solidifying.

Coating of the media channels with HUVECs

On d4 of iPSC-HLC on-chip culturing, the HCM medium was changed into mixed culture medium (HCM:EGM2) and the chips' media channels were coated with HUVECs. The HUVECs were cultured in T-75 bottles in EGM2 and used at passages 6-7. The cells were detached with TrypLE Select (Gibco), collected, and resuspended in the mixed culture medium for cell counting. After counting the cells, the cell suspension was diluted into a concentration of 2 million cells/ml. In preparation for the coating, the chip media channels were first washed with PBS before adding 60 μg/ml fibronectin solution (Millipore) to the empty channels. The chips were placed into an incubator for 60 minutes. Afterwards they were washed with PBS and 50 μl of

mixed culture medium was added to each medium port. Left-side media channels were coated first. To create flow in the medium channel, 20 µl of the mixed culture medium was added to the top port right before slowly pipetting 10 µl of HUVEC cell suspension directly into the medium channel, through the medium inlet. The cell distribution was observed until the flow decreased and the process was repeated to the bottom end of the channel. Next the chips were turned 90° so that they were standing on their sides and the cells would attach to the gel-medium interface on the right side of the media channels. The chips were placed in an incubator like this for 2 h before repeating the seeding process for the right-side media channels. After the media channels were coated with HUVECs on both sides, the medium levels in each port were checked and adjusted to the usual 70 µl on top and 50 µl on the bottom.

Staining protocol for the microfluidic chips

Cells were fixed by adding 4% paraformaldehyde (PFA) and incubating the chips in room temperature for 1h. Next, they were washed twice with PBS (Gibco) and stored at +4°C until the start of the staining protocol. The full immunofluorescence staining protocol is presented in Table S5 and information about the primary and secondary antibodies in Table S4. For the short 5-minute-long incubations, a gravitational flow was established through the media channels, and during the longer incubations the flow was directed through the gels by adding more liquid volume on the left-side channel than the right side. After the staining protocol was finished, the chips were stored light-protected at +4°C. The samples were imaged with Olympus IX51 Fluorescence Microscope (Olympus Corporation).

Supplementary results

Preliminary biomaterial tests with HepG2 and HUVECs

Both 3:2 and 2:1 Geltrex-to-cell suspension ratios behaved similarly during the preliminary biomaterial tests with HepG2 and HUVECs, and no visible differences were observed between the gels. The distribution of the gel inside the channel was even, cells in the gel stayed in 3D, and disintegration of the gel was only observed at the ends of the channels where the HepG2 cells proliferated aggressively. Compared to fibrin or collagen I, the cells proliferated a lot more when embedded in Geltrex. However, the higher Geltrex-to-cell ratio 2:1 was more difficult to handle and pipette into the gel channels without forcing the material into the flanking media channels, especially with the higher cell concentrations. The lowest cell density, 8 million cells/ml, was too low while the two higher concentrations, 12 and 20 million cells/ml, offered a good HepG2 cell density in both Geltrex-to-cell ratios 3:2 and 2:1. However, due to very high number of cells needed to reach 20 million cells/ml, the 12 million cells/ml concentration was chosen for the subsequent chip experiments.

In fibrin tests the gel distribution was even in the gel channel and the cells stayed viable and in 3D throughout the experiments for all fibrinogen concentrations. The higher concentration gels (20 and 10 mg/ml) showed no signs of degradation during the experiment, but the lowest concentration (5.0 mg/ml) allowed the microspheres to enter the gel channel. However, the microspheres did not cross the gel channel to the opposite medium channel. We did not observe differences between the two higher concentration gels.

A visible difference between the different collagen I concentrations was seen just hours after seeding the cells onto the chips. In the lowest gel concentration (0.5 mg/ml) the cells were less rounded and there were air bubbles forming in the media channels. During the experiment, dead cells flushed out of the gel channel into the media channels. This was not observed in the higher gel concentrations. However, all three gel concentrations were visibly different from each other throughout the experiment, even though the cells stayed mostly in 3D in all of them. When the viability of the cells was assessed with a live/dead stain, all three Collagen I concentrations had more dead cells compared to the Geltrex and fibrin chips, with the highest concentration (5.0 mg/ml) having slightly more than the other two. When the gel integrity was tested, the microspheres passed straight through the 0.5 mg/ml gel, implying that the material had degraded during the experiment as opposed to the gels with higher collagen I concentrations, which didn’t let any microspheres through.

In experiments with HUVECs and Puramatrix, all cells were dead by day 3 of on-chip culturing, even though the gel seemed intact until day 6. Thus, we decided not to go

forward with Puramatrix in the chip experiments and concluded that the cell death was probably due to the very acidic nature of the material. In the spheroid experiments, cell death was avoided as the spheroids were cultured in large volume of medium, which was changed twice during the first hour after making the spheroids. We noticed in our spheroid experiments that the sooner the first medium change was, the better the cells survived.

Multilineage spheroid preliminary medium tests

We first performed a preliminary medium test with the multilineage spheroids (iPSC-HLCs + HUVECs + hASCs) to see which combination of HCM and EGM2 media would best support the hepatocyte maturation as well as the survival of the other two cell types. As a reference, we had iPSC-HLC monoculture spheroids in all three medium combinations. In the HLC monoculture spheroids, the gene expression levels of *AFP, ALB, APOB* and *CYP3A5* were highest in the 100% HCM at d12 compared to d7, *ApoA1* and *ASGPR1* levels were highest with the 75:25 medium mix at both time-points as compared to d7 100% HCM. (Fig S8) When comparing the gene expression levels of the multilineage spheroids to the iPSC-HLC monoculture spheroid d7 100% HCM, the level of all studied genes was lower at both d7 and d12 in all media combinations, except *ALB* which was at the same level in both spheroid types at d12 in 100% HCM medium (Fig S8). Thus, the 100% HCM seemed to work best for the multilineage spheroids when compared to the iPSC-HLC spheroids.

At d7 the endothelial cell marker *CD31* expression level in multilineage spheroids was higher in 50:50 and 75:25 media compared to 100% HCM but by d12 the expression levels were negligible in multilineage spheroids, regardless of the medium. We decided to continue the experiments with the 100% HCM medium as it best supported the iPSC-HLC liver-like phenotype.

Supplementary Tables

Table S1. Details of the experiments with spheroids in cell culture inserts and vasculature in the bottom of each well. In all experiments a 50:50 mix of HCM:EGM2 with growth factors (GF) was used.

Exp	HUVEC:hASC ratio	Vessel maturity	Insert type	hepatic diff. day at spheroid d0
**SV1**	5:1	d7	Millicell, 1,0µm PET	d11
**SV2**	5:4	d4	Brand, 0,4µm PC	d11
**SV3**	5:1	d5	Brand, 0,4µm PC	d10

Abbreviations: HCM, hepatocyte culture medium; EGM2, endothelial growth medium 2; GF, growth factor; HCM supplemented with 25 ng/ml HGF (hepatocyte growth factor) and 20 ng/ml OSM (oncostatin M). HUVECs used were GFP-tagged in all but experiment SV3.

Table S2. Details of the biomaterial tests performed with HepG2 cells on the microfluidic chip (AIM Biotech).

Number of Chips	Biomaterial	Biomaterial concentration or ratio	Medium	Cell concentration
6	Geltrex	GT:CS = 3:2<br>GT:CS = 2:1	DMEM	20 million cells/ml<br>12 million cells/ml<br>8 million cells/ml
4	Fibrin*	20 mg/ml<br>10 mg/ml<br>5.0 mg/ml	DMEM	12 million cells/ml
3	Collagen I (5mg/ml)	2.0 mg/ml<br>1.0 mg/ml<br>0.5 mg/ml	RPMI 1640	12 million cells/ml

Abbreviations: GT, Geltrex; CS, cell suspension; *Fibrin with 10 mg/ml fibrinogen and 2IU/ml Thrombin

Table S3. Immunocytochemical staining protocol for 2D samples.

Phase	Time	Solution
Blocking (RT)	45min	10% NDS, 0,1% TritonX-100 and 1% BSA in PBS (Gibco) (v/v)
1st wash (RT)	1x 5min	1% NDS, 0,1% TritonX-100 and 1% BSA in PBS (v/v)
Primary antibodies (4°C)	Overnight	Diluted in 1% NDS, 0,1% TritonX-100 and 1% BSA in PBS
2nd wash (RT)	3x 5min	1% BSA in PBS (v/v)
Secondary antibodies (RT)	1h	Diluted in 1% BSA in PBS (v/v)
3rd wash (RT)	3x 5min	1x PBS
4th wash (RT)	2x 5min	PB

Abbreviations: NDS, Normal donkey serum; BSA, Bovine serum albumin; PBS, Phosphate-Buffered Saline; PB, Phosphate Buffer; RT, Room temperature

Table S4. Primary and secondary antibodies used for staining 2D samples, 3D spheroid and microfluidic chip (AIM Biotech) cultures.

Primary antibody	Origin	Manufacturer	Dilution
A1AT	Rabbit	Abcam	1:400
AFP	Rabbit	Dako	1:400
Albumin	Mouse	R&D	1:100
CD31	Mouse	Dako	1:200
CK19	Mouse	Invitrogen	1:100
MRP2	Rabbit	Abcam	1:100
SOX9	Rabbit	Invitrogen	1:400
**Secondary antibody**	**Origin**	**Manufacturer**	**Dilution**
Anti-rabbit IgG 488	Donkey	Invitrogen	1:300
Anti-mouse IgG 568	Donkey	Invitrogen	1:300

Abbreviations: A1AT, Alpha-1 antitrypsin; AFP, Alpha-fetoprotein; CD31, cluster of differentiation 31; CK19, Cytokeratin 19; MRP2, Multidrug resistance-associated protein 2

Table S5. Steps of the immunofluorescence protocol used to stain the cells cultured in the microfluidic chips (AIM Biotech).

Stage	Incubation time and temperature	Solution
**Blocking**	Overnight @+4°C	10% NDS, 0.1% Triton-X, 1% BSA in PBS
**1st wash**	1x 5 min; RT	1% NDS, 0.1% Triton-X, 1% BSA in PBS
**Primaries**	Overnight @+4°C	In 1st wash solution
**2nd wash**	1x 5 min, 3x 2 h; RT	1% BSA in PBS
**Secondaries**	Overnight @+4°C	In 2nd wash solution
**3rd wash**	1x 5 min, 1x 1 h; RT	PBS
**DAPI**	1h; RT	1:1000 in PBS
**4th wash**	1x 5 min, 2x 1 h; RT	PBS

Abbreviations: NDS, Normal Donkey Serum (Millipore); BSA, Bovine Serum Albumin; PBS, Phosphate Buffered Saline; DAPI, 4' 6-diamidino-2-phenylindole dihydrochloride (Sigma-Aldrich); RT, room temperature

Table S6. The iPSC-HLC and iPSC-HLC+HUVEC spheroid volumes extracted from segmented 3D OPT reconstructions.

Spheroid day	Spheroid type (No. of parallel samples)	Average spheroid volume ± SD (µl)
**d0**	iPSC-HLC (3)	2.0 ± 0.2
	iPSC-HLC+HUVEC (3)	2.1 ± 0.1
**d14**	iPSC-HLC (2)	1.5 ± 0.3
	iPSC-HLC+HUVEC (2)	1.6 ± 0.4
**d20**	iPSC-HLC (1)	0.4
	iPSC-HLC+HUVEC (3)	0.3 ± 0.2

Supplementary Figures

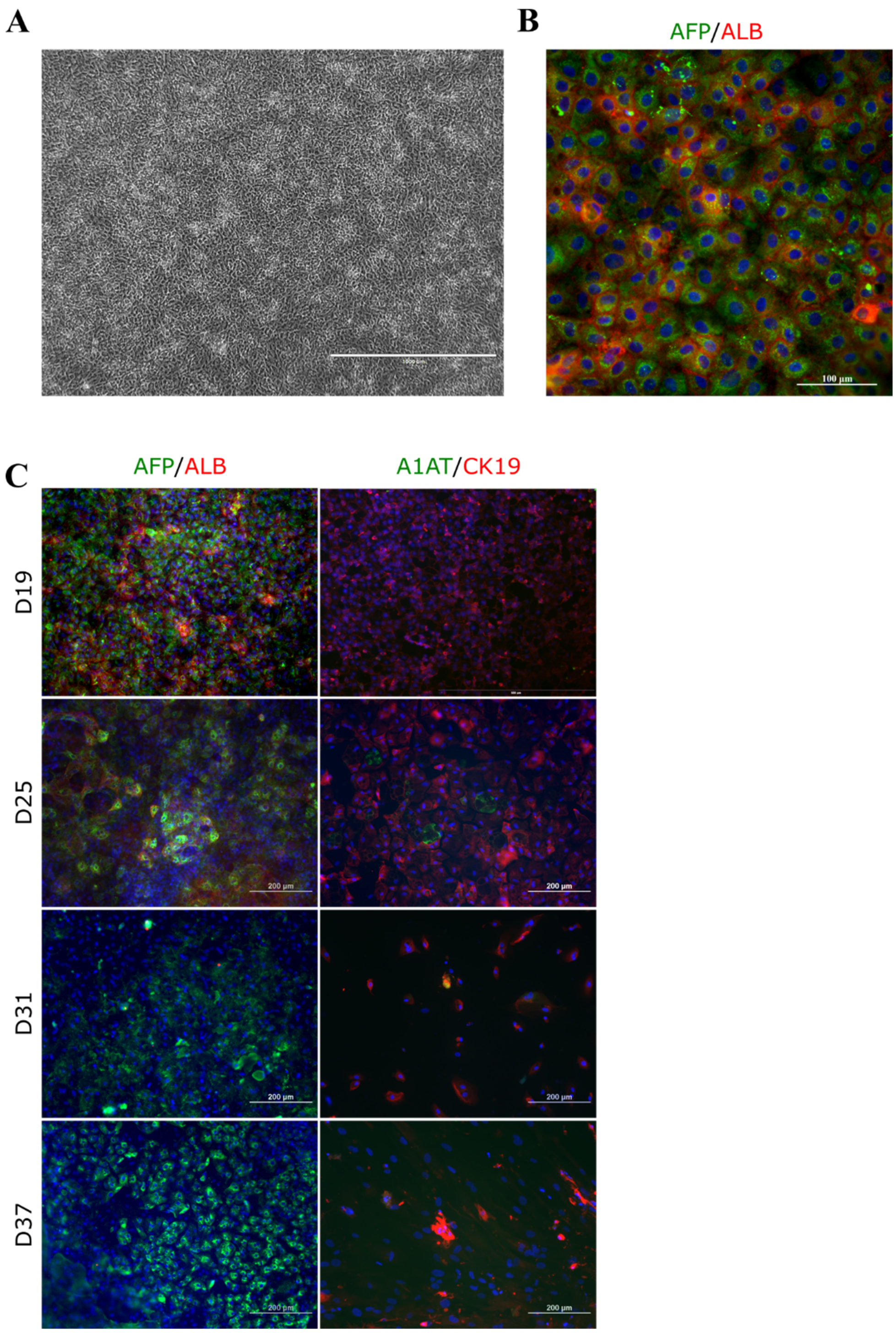

Figure S1. Cell morphology and protein expression in iPSC-HLC 2D cultures. A) Bright field image of differentiated iPSC-HLCs on d19 of 2D culture, scale bar 1000 µm. B) Representative image of ALB-AFP stained iPSC-HLCs on d19 of 2D culture, scale bar 100µm. C) Fixed 2D cultures were stained at differentiation days 19, 25, 31 and 37. The samples were imaged with Olympus IX51 Fluorescence microscope (Olympus Corporation). The images show hepatocyte markers alpha fetoprotein

(AFP), albumin (ALB), Alpha 1 antitrypsin (A1AT), and hepatoblast/cholangiocyte marker cytokeratin 19 (CK19). Nuclei were stained with DAPI (blue). Scale bars 200 μm.

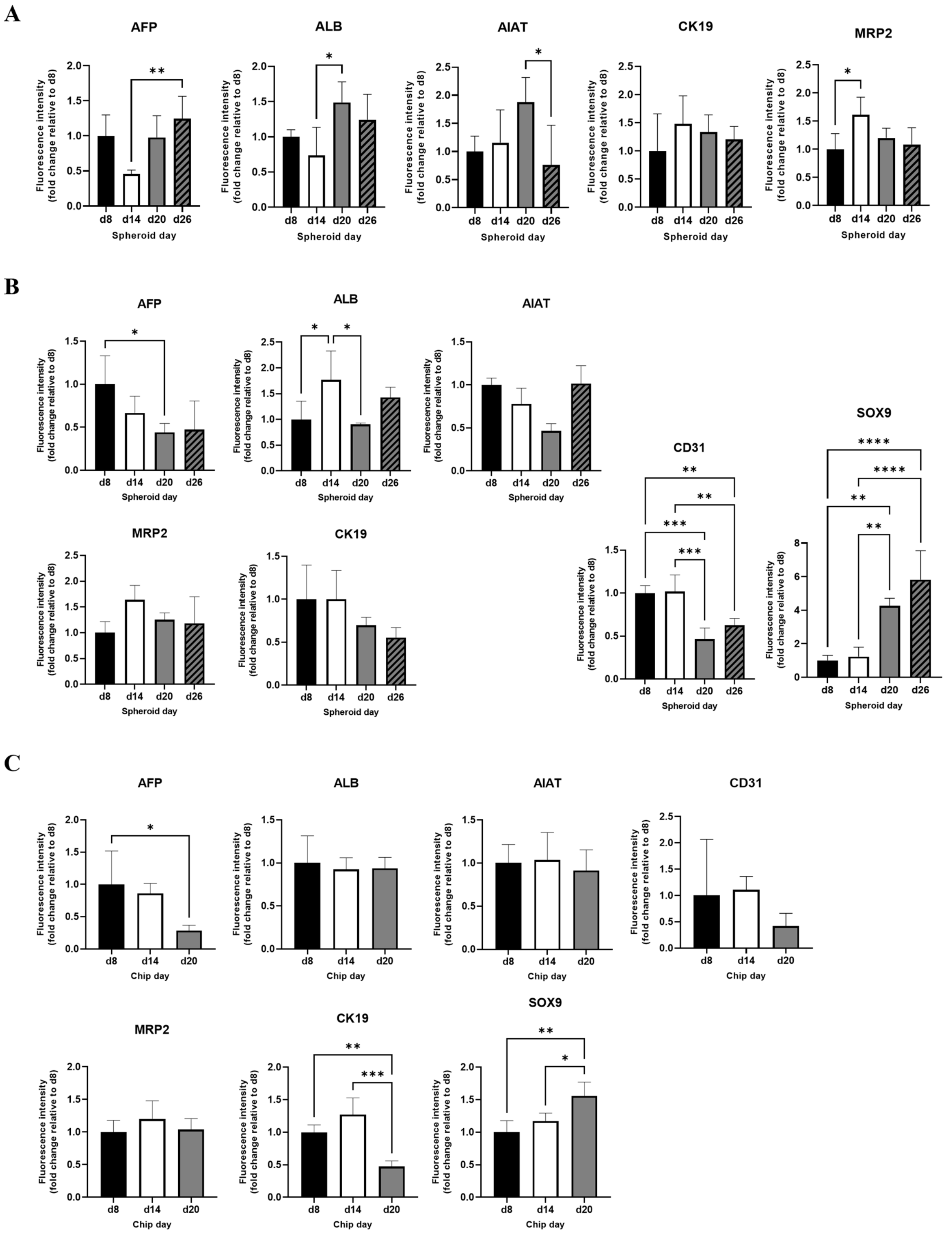

Figure S2. Corresponding fluorescence intensity quantification of spheroid and microfluidic chip culture images presented in Figures 3A, 8 and 10. A) Corresponding fluorescence intensity quantification for iPSC-HLC spheroid sections presented in Figure 3A. B) Corresponding fluorescence intensity quantification for multilineage

spheroid sections presented in Figure 8. C) Corresponding fluorescence intensity quantification for multilineage microfluidic chip cultures presented in Figure 10. Fold changes for all markers are shown as relative to their respective culture day d8. Data represent the mean ±SD from four measured fields in the images; 1way ANOVA followed by Tukey's multiple comparison test; * < 0.05, **<0.01, ***<0001. AFP, alpha-fetoprotein; ALB, albumin; A1AT, alpha 1 antitrypsin; CK19, cytokeratin 19; MRP2, multidrug resistance-associated protein 2; CD31, cluster of differentiation 31 (endothelial cell marker).

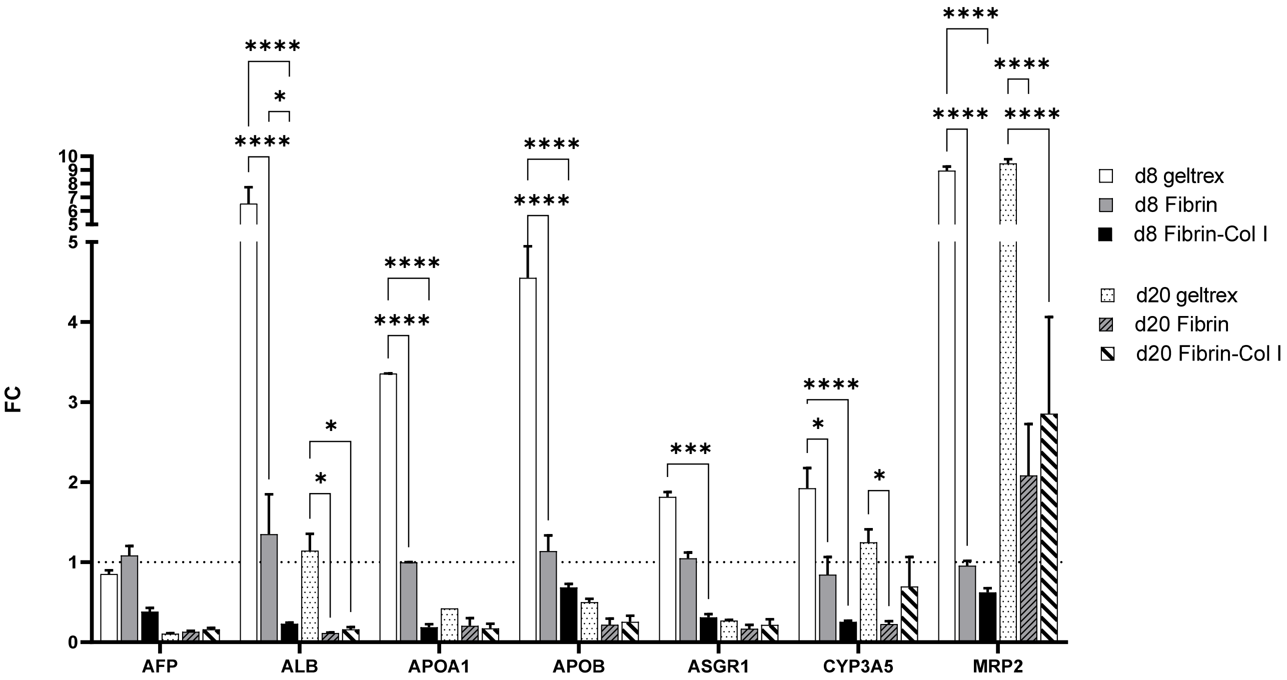

Figure S3. Expression of liver-related genes in iPSC-HLCs cultured on-chip for 8 or 20 days in different biomaterials to support 3D growth. *GAPDH* was used as the endogenous control and fold change is calculated relative to d8 Fibrin samples. 2way ANOVA and Tukey's multiple comparison test were performed, p-value * <0.05, **<0.01, ***<0.001, ****<0.0001. ColI, Collagen I

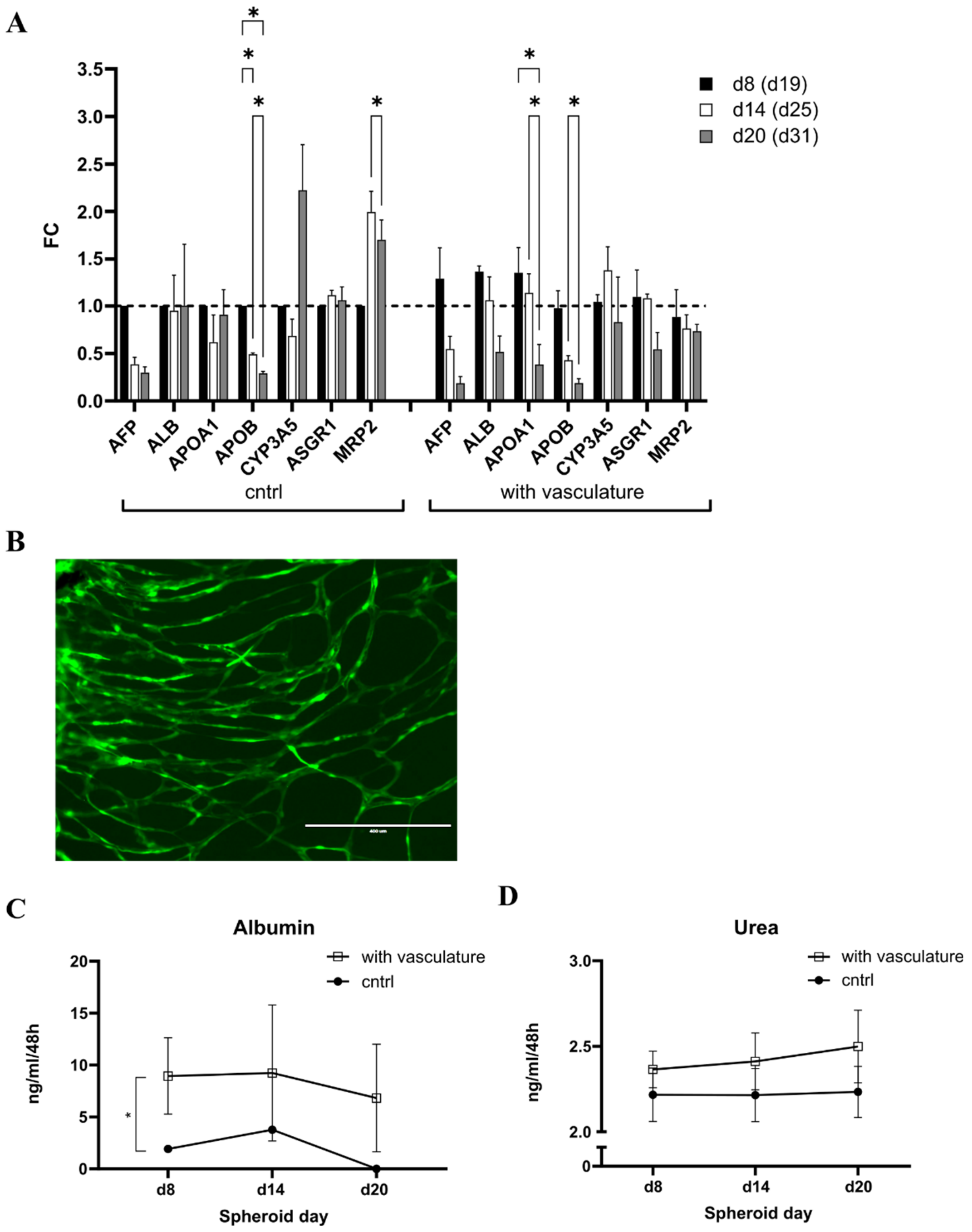

Figure S4. Comparing the iPSC-HLC spheroids cultured in inserts with or without vasculature. A) Gene expression levels were measured from three separate experiments and are expressed as mean +SEM (two biological replicates/ time-point run in triplicate). *GAPDH* was used as an endogenous control, fold change (FC) calculated relative to d8 cntrl spheroids. *p<0.05. B) Representative image of vasculature in the bottom of a cell culture well, scale bar 400μM. C) Albumin and D) urea production by iPSC-HLC spheroids cultured in cell culture inserts with or without vasculature (cntrl) in the bottom of the cell culture well. Values are mean ± SD (for ALB overall p<0.01; *p<0.05).

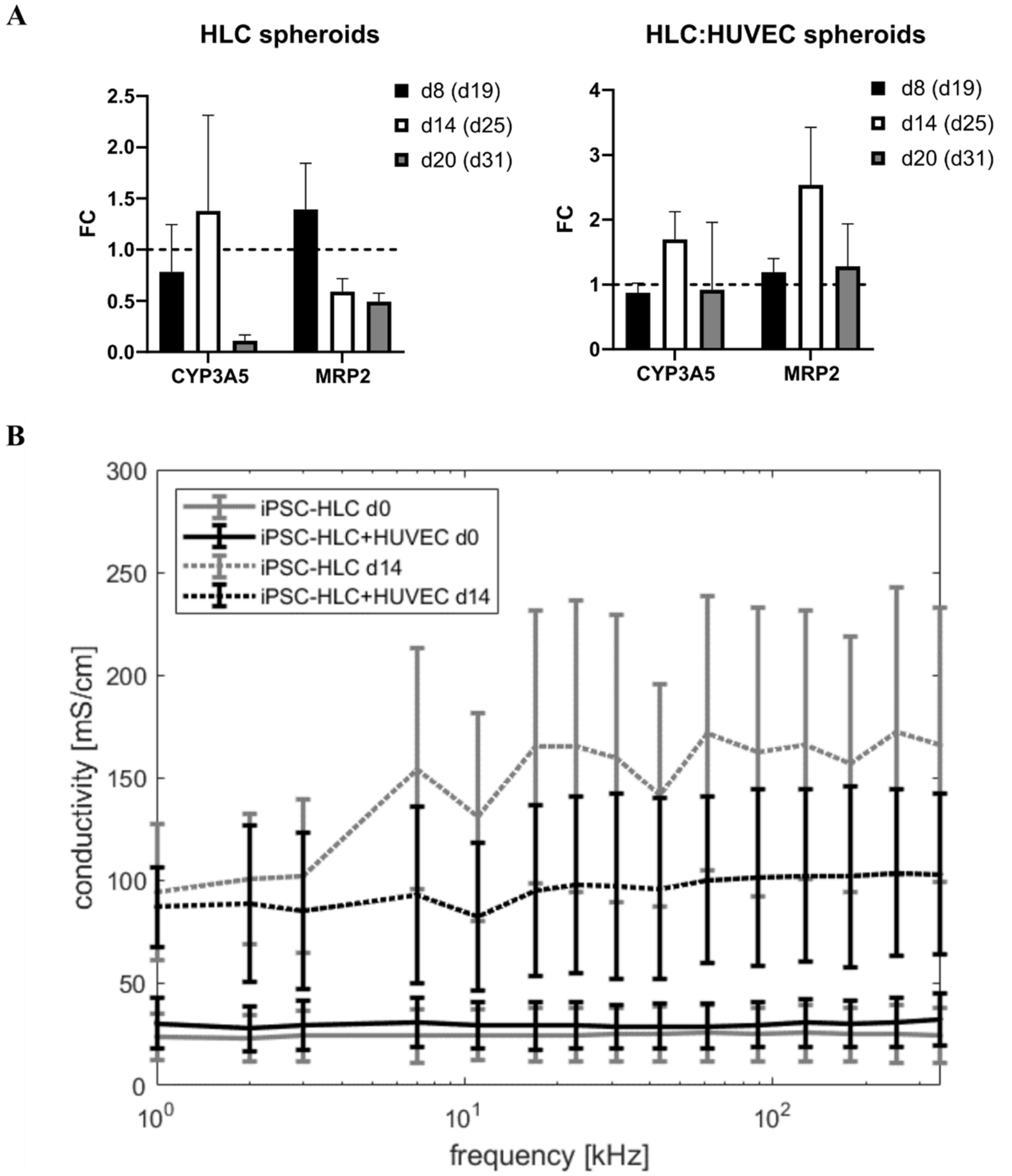

Figure S5. Gene expression and electrical conductivities of iPSC-HLC and iPSC-HLC+HUVEC spheroids. A) Expression of *CYP3A5* and *MRP2* did not change statistically significantly during spheroid culture from spheroid d8 (differentiation d19) to d20 (differentiation d31) in either spheroid type. B) The electrical conductivities increased from spheroid day d0 to d14. Conductivities are extracted from OPT-EIT 3D reconstructions. Data is shown as mean ± SD and n=3 on d0 samples and n=2 on d14 samples.

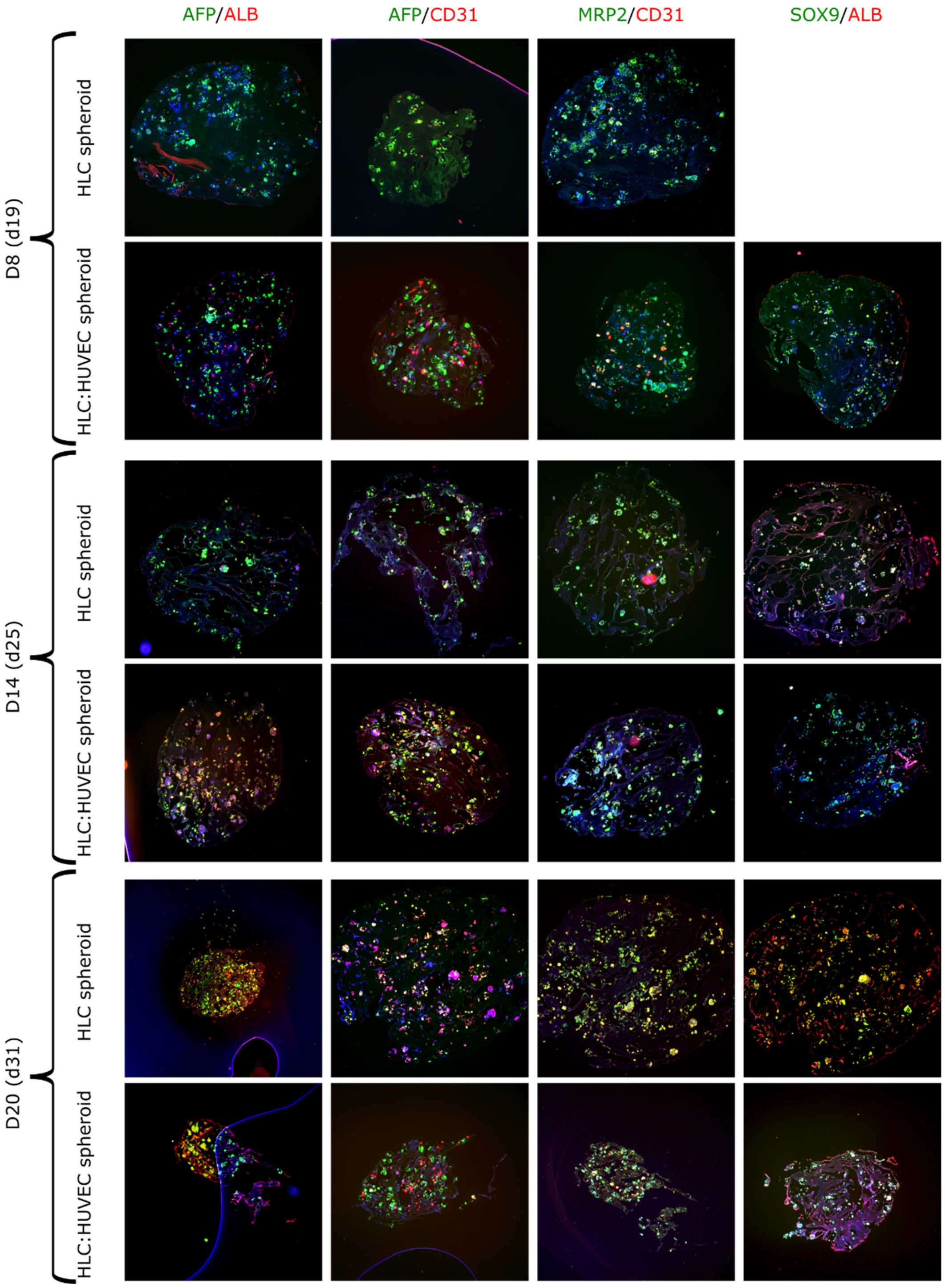

Figure S6. Expression of hepatic and endothelial marker proteins in the iPSC-HLC monoculture and iPSC-HLC+HUVEC spheroids at d8, 14 and 31, corresponding to differentiation days 19, 25 and 31, respectively. All spheroids were cultured in mixed medium (HCM:EGM2), fixed, cut into sections, stained and imaged with Olympus IX51 Inverted Phase Contrast Fluorescence Microscope (Olympus Corporation, Hamburg, Germany). ALB, albumin; AFP, alpha-fetoprotein; MRP2, multidrug resistance protein 2; SOX9 and CK19, cytokeratin 19 (hepatocyte progenitor/cholangiocyte markers); CD31, cluster of differentiation 31 (a marker for endothelial cells, i.e. HUVECs)

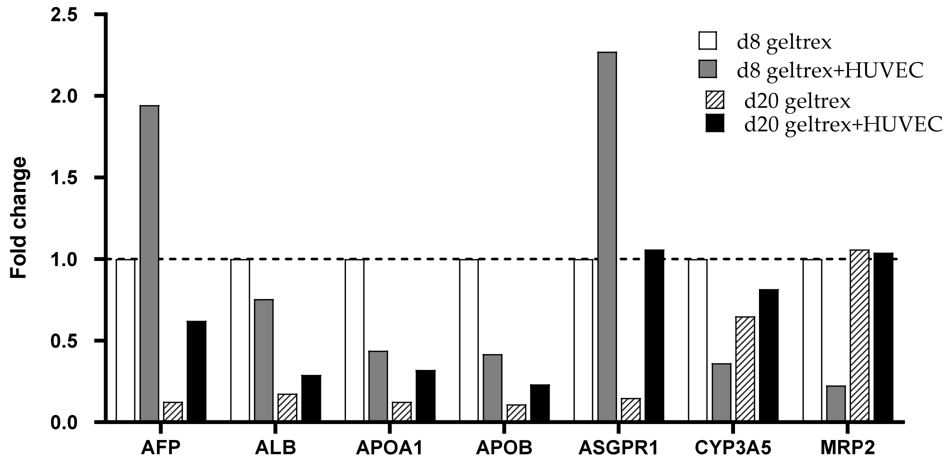

Figure S7. Gene expression levels of liver-specific genes in iPSC-HLCs monoculturechips and in chips with iPSC-HLCs and HUVECs mixed and plated in the gel channel of the microfluidic chip (AIM Biotech).

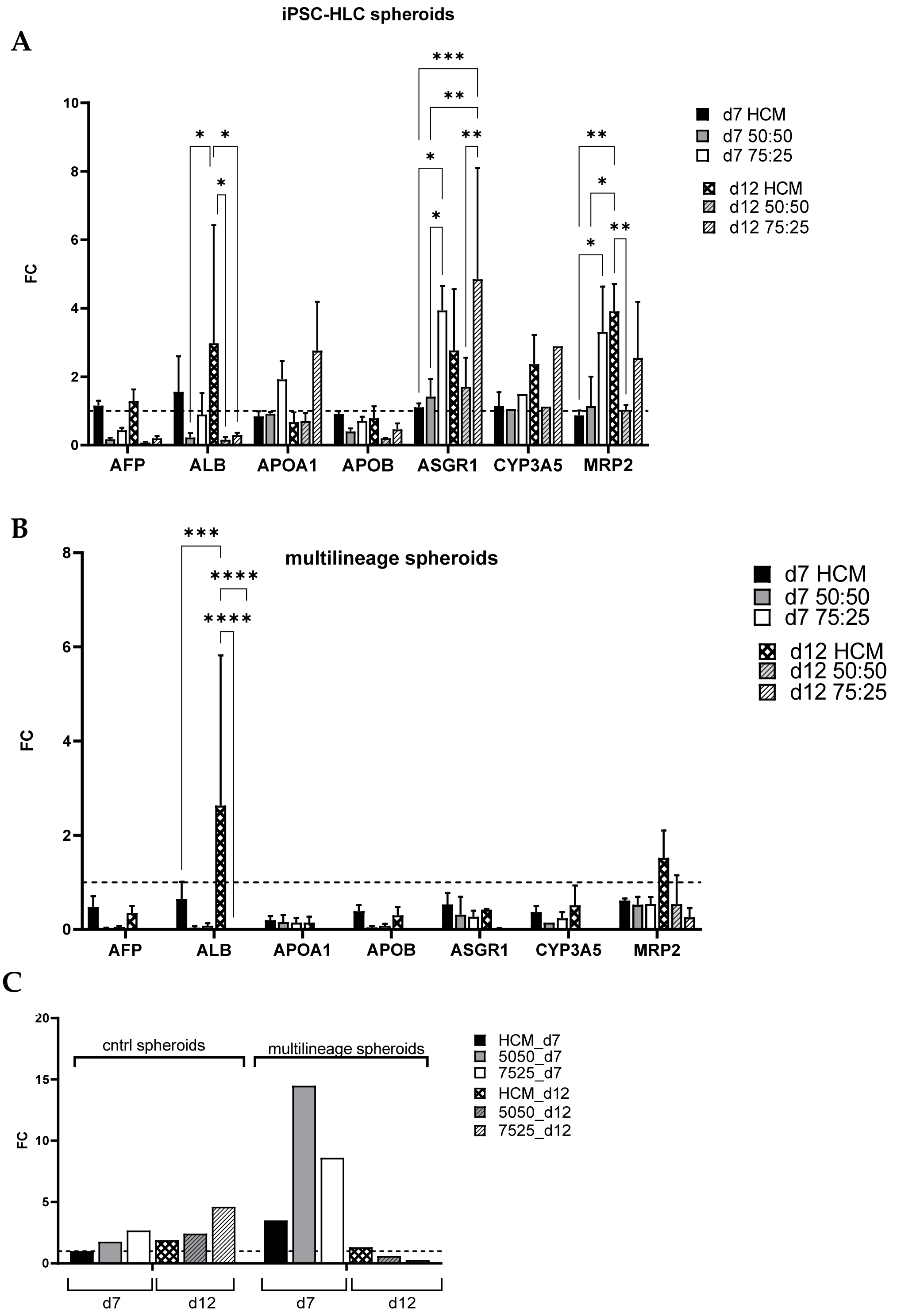

Figure S8. Gene expression of iPSC-HLC and multilineage spheroids in different cell culture media mixes. A) The iPSC-HLC spheroids and B) multilineage spheroids gene expression fold change using iPSC-HLC spheroid d7 HCM as reference; C) CD31 expression in both iPSC-HLC and multilineage spheroids when compared to iPSC-HLC day 7 HCM sample. Media: HCM, 100% hepatocyte growth medium; 50:50, 50% HCM and 50% EGM2; 75:25, 75% HCM and 25% EGM2. 2way ANOVA and Tukey's multiple comparison test were performed, p-value * <0.05, **<0.01, ***<0.001, ****<0.0001

Supplementary videos

The spheroid videos show examples of the raw OPT data on spheroid days 0, 14 and 20, corresponding to differentiation days 11, 25 and 31.

Supplementary OPT videos:

V**ideo S1**. iPSC-HLC spheroids at d0.

Video S2. iPSC-HLC + HUVEC spheroids at d0.

Video S3. iPSC-HLC spheroids at d14.

Video S4. iPSC-HLC+HUVEC spheroids at d14

Video S5. iPSC-HLC + HUVEC spheroids at d20.

Video S6. SPIM imaging of a multilineage spheroid at d14 stained with CD31 (red) and AFP (green).
