# Supplementary figures and images for: Improvements in Maturity and Stability of 3D iPSC-Derived Hepatocyte-like Cell Cultures
Source: Cells. 2023 Sep 27;12(19):2368. doi: 10.3390/cells12192368 (PMC10571736; doi:10.3390/cells12192368)

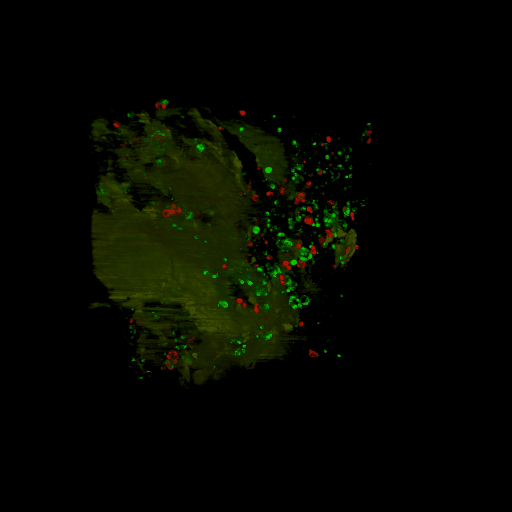

Supplement: Supplementary file 1 [file cells-12-02368-s001.zip › Video S6_multilineage spheroid__d14_.gif]
